# Characterizing temporal and global host innate immune responses against SARS-CoV-1 and -2 infection in pathologically relevant human lung epithelial cells

**DOI:** 10.1371/journal.pone.0317921

**Published:** 2025-01-28

**Authors:** Vivian Y. Tat, Aleksandra K. Drelich, Pinghan Huang, Kamil Khanipov, Jason C. Hsu, Steven G. Widen, Chien-Te Kent Tseng, George Golovko

**Affiliations:** 1 Department of Pathology, The University of Texas Medical Branch, Galveston, Texas, United States of America; 2 Department of Microbiology & Immunology, The University of Texas Medical Branch, Galveston, Texas, United States of America; 3 Department of Pharmacology & Toxicology, The University of Texas Medical Branch, Galveston, Texas, United States of America; 4 Department of Biochemistry & Molecular Biology, The University of Texas Medical Branch, Galveston, Texas, United States of America; Qinghai University, CHINA

## Abstract

Severe acute respiratory syndrome coronavirus-1 (SARS-CoV-1) and -2 (SARS-CoV-2) are beta-coronaviruses (β-CoVs) that have caused significant morbidity and mortality worldwide. Therefore, a better understanding of host responses to β-CoVs would provide insights into the pathogenesis of these viruses to identify potential targets for medical countermeasures. In this study, our objective is to use a systems biology approach to explore the magnitude and scope of innate immune responses triggered by SARS-CoV-1 and -2 infection over time in pathologically relevant human lung epithelial cells (Calu-3/2B4 cells). Total RNA extracted at 12, 24, and 48 hours after β-CoVs or mock infection of Calu-3/2B4 cells were subjected to RNA sequencing and functional enrichment analysis to select genes whose expressions were significantly modulated post-infection. The results demonstrate that SARS-CoV-1 and -2 stimulate similar yet distinct innate antiviral signaling pathways in pathologically relevant human lung epithelial cells. Furthermore, we found that many genes related to the viral life cycle, interferons, and interferon-stimulated genes (ISGs) were upregulated at multiple time points. Based on their profound modulation upon infection by SARS-CoV-1, SARS-CoV-2, and Omicron BA.1, four ISGs, i.e., bone marrow stromal cell antigen 2 (*BST2*), Z-DNA Binding Protein 1 (*ZBP1*), C-X-C Motif Chemokine Ligand 11 (*CXCL11*), and Interferon Induced Transmembrane Protein 1 (*IFITM1*), were identified as potential drug targets against β-CoVs. Our findings suggest that these genes affect both pathogens directly and indirectly through the innate immune response, making them potential targets for host-directed antivirals. Altogether, our results demonstrate that SARS-CoV-1 and SARS-CoV-2 infection induce differential effects on host innate immune responses.

## Introduction

Severe acute respiratory syndrome coronavirus (SARS-CoV-1) and SARS-CoV-2 are beta-coronaviruses (β-CoVs) that have emerged in the past 20 years, causing significant morbidity and mortality worldwide. SARS-CoV-1, which is the original SARS-CoV, was the causative agent of the 2003 SARS outbreak, with a case fatality rate of approximately 10% [1]. SARS-CoV-2 is responsible for the coronavirus disease (COVID-19) pandemic. As of March 12, 2024, more than 704.0 million people have been infected by SARS-CoV-2, and more than 7.0 million have died of COVID-19 [2]. Severe disease is often caused by an excessive inflammatory response in the lungs, leading to cytokine release syndrome [3]. This in turn contributes to organ damage, acute respiratory distress syndrome, diffuse alveolar damage, and potentially death [3,4].

The innate immune response is activated when pathogens such as SARS-CoV-1 or -2 enter the host. Inherited cytosolic pattern recognition receptors (PRRs) expressed within airway epithelia sense virus-specific pathogen-associated molecular pattern molecules (PAMPs) to trigger type I and III interferons (IFNs), along with other inflammatory mediators, eventually leading to the activation of interferon-stimulated genes (ISGs) [5]. ISGs exert broad antiviral activities, such as suppression of viral replication, enhanced pathogen detection, promotion of innate immune signaling, and targeting of pathways and functions required for the viral life cycle.

However, β-CoVs have evolved sophisticated strategies to delay or even avoid induction of the host’s innate immune response to successfully establish their infection [6,7]. As a result, the failure to induce an effective response in a timely fashion could cause early and overwhelming viral replication and dysregulated and often exuberant inflammatory responses [8].

Though the innate immune response can be impeded by β-CoVs, the host is still able to partially defend itself [7]. Previous work from our lab explored the innate immune response of pathologically relevant human bronchial epithelial cells (Calu-3/2B4) to SARS-CoV-1 and found that transcription factors, pathways involving chemokines, and the innate immune and inflammatory response were upregulated following infection [9]. Type I and III IFNs and ISGs were also activated to defend the host [9]. Highlighting the importance of the innate immune response in defending the host against β-CoVs infection, patients with severe COVID-19 have low expression of ISGs [10–12].

Many publications have examined SARS-CoV-1 and -2 gene expression profiles; however, few have directly compared the temporal and global host profile induced by SARS-CoV-2 with that of SARS-CoV-1 infection [13,14]. Additionally, those studies focused on exploring different signaling pathways and/or used different cell models, such as Calu-3 cells, which has lower expression of the human angiotensin converting enzyme 2 (ACE2) receptor [13,14]. Thus, to our knowledge, there has yet to be any published studies comparing ISGs elicited by SARS-CoV-1 and SARS-CoV-2 infection in Calu-3/2B4 cells. Our goal is to focus on the similarities of the innate immune response induced by SARS-CoV-1- and -2-infection to identify druggable targets for both of these highly pathogenic β-CoVs.

Through our work, we identified genes and pathways involved in the lung pathogenesis of SARS and COVID-19 through systems biology and experimental approaches. Specifically, we found that many ISGs, as components of canonical pathways related to PRRs, IFN signaling, and response to viral infection, are upregulated over time to varying extents. Among those upregulated ISGs, we selected four, *BST2*, *ZBP1*, *CXCL11*, and *IFITM1*, for future exploration as druggable targets to attenuate viral infection. Thus, we expand on the innate signaling pathways triggered by SARS-CoV-1 and SARS-CoV-2 infection and explore potential therapeutic targets to combat β-CoVs.

## Materials and methods

### Cells

Calu-3/2B4 cells were grown in Eagle’s 1X Minimal Essential Medium (MEM) [Corning, Cat. No. 10-010-CV] supplemented with 20% fetal bovine serum (FBS) [GIBCO, Cat. No. 10437–028], 0.01% L-Glutamine [GIBCO, Cat. No. 25030–164], and 0.01% Penicillin-Streptomycin [GIBCO, Cat. No. 15140–122]. Vero E6 cells were grown in Eagle’s 1X MEM supplemented with 10% FBS, 0.01% L-Glutamine, and 0.01% Penicillin-Streptomycin [15].

### Viruses

The Urbani strain of SARS-CoV-1, kindly provided by Dr. T. G. Ksiazek at UTMB, was used throughout this study. The original stock of SARS-CoV-1 was subjected to two additional passages in Vero E6 cells using MEM supplemented with 2% FBS, 0.01% L-Glutamine, and 0.01% Penicillin-Streptomycin (2-MEM). A viral stock with a titer of ~ 7.5 x 10^6^ 50% tissue culture infectious doses per milliliter (TCID_50_/mL) was generated and stored at -80°C.

The USA-WA1/2020 strain of SARS-CoV-2 (WA-1), kindly provided by Dr. Natalie Thornburg at the Centers for Disease Control (CDC), Atlanta, GA, and the World Reference Center for Emerging Viruses and Arboviruses (WRCEVA), was used throughout this study. The original stock of SARS-CoV-2 was cultured in 2-MEM and passaged once in Vero E6 cells to generate working viral stocks, which were stored at -80°C. The working viral stocks used throughout this study were titrated at ~ 5 × 10^6^ TCID_50_/mL by the standard TCID_50_ assay in Vero E6 cells.

The Omicron BA.1 strain of SARS-CoV-2 (Omicron BA.1), kindly provided by WRCEVA, was used throughout this study. The original stock of Omicron was cultured in 2-MEM and passaged one more time in Vero E6 cells to generate working viral stocks, which were stored at -80°C. The working viral stocks used throughout this study were titrated at 1 × 10^6^ TCID_50_/mL by the standard TCID_50_ assay in Vero E6 cells.

All infectious SARS-CoV-1 and SARS-CoV-2 experiments were conducted at UTMB in an approved biosafety level 3 laboratory.

### Tissue culture infectious dose (TCID_50_) assay

To measure viral yields, we took 50 μL of cell-free viral samples into 450 μL of 2-MEM, then performed serial dilutions from 10^−1^ to 10^−8^ [15]. Afterwards, we aliquoted 100 μL of the dilution into a 96-well plate of confluent Vero E6 cells, with four wells per dilution. The 96-well plate was then incubated at 37°C at 5% CO_2_ to visualize cytopathic effect (CPE). After three days, wells with CPE were considered “dead”, and the number of viable virus particles were calculated and quantified using TCID_50_/mL.

### RNA isolation

In addition to collecting supernatant for viral titration and confirming that Calu-3/2B4 cells were permissive to both β-CoVs infection, we also harvested the monolayers of infected and mock-infected Calu-3/2B4 cells, which were extracted for total RNA at each time point. To isolate RNA, we utilized the Invitrogen™ TRIzol™ Reagent for RNA extraction followed by Direct-Zol™ RNA Miniprep kit for RNA cleanup. TURBO DNA-*free*™ kit was then used for post-RNA extraction DNase digestion. Afterwards, we converted the extracted RNA into cDNA using the iScript™ cDNA Synthesis Kit. The extracted RNA was then converted into 500 or 600 nanograms per microliter (ng/μL) of cDNA using the iScript™ cDNA Synthesis Kit. The cDNA was then used for RNA-Seq or RT-qPCR.

### RNA-Sequencing (RNA-Seq) analysis

RNA-Seq was performed by the UTMB Next Generation Sequencing (NGS) Core Facility using the Illumina NextSeq 550 platform with polyA+ mRNA sequencing libraries on a single 400 million read. Our comparisons were SARS-CoV-1 versus Mock, SARS-CoV-2 versus Mock, and SARS-CoV-2 versus SARS-CoV-1.

Normalization of the data was performed using DESeq2’s median of ratios methods. Afterwards, quality filtering and adapter trimming was performed using trimmomatic-0.39 with a quality cutoff at QScore30. To map the reads, Spliced Transcripts Alignment to a Reference (STAR) version 2.7.10a was utilized. The hg38 genome was used to build the index, and to quantify the reads mapping to the genes, the STAR-quantMode GeneCounts option was used. To estimate differential gene expression and measure log fold changes, the R v4.1.3 DESeq2 software package, version 1.32.0, along with the lfcShrink function and the ashr package, were used. Negative binomial generalized linear models were used for statistical testing. Our results were deposited in Gene Expression Omnibus (GEO), and the dataset accession number is GSE255647.

### Functional enrichment analysis

We utilized several bioinformatics tools and applications to analyze the results of RNA-Seq. QIAGEN Ingenuity Pathway Analysis (IPA) is a versatile web-based software application used for the analysis, integration, and interpretation of complex biological data from experiments such as gene expression, miRNA, and proteomics [16]. It offers data analysis and interpretation by placing experimental results within the context of biological systems, aided by a comprehensive, manually curated database. To calculate the p-values of the canonical pathways, a right-tailed Fisher’s Exact Test was utilized [17].

We also utilized the Cytoscape application ClueGO, a tool to analyze the functional enrichment of genes to create and visualize networks of terms and pathways based on their corresponding function [18]. We integrated three gene ontology platforms: Gene Ontology (GO) [19], Kyoto Encyclopedia of Genes and Genomes (KEGG) [20], and Reactome [21].

For further validation, we utilized GeneAnalytics™ to identify and analyze gene expression patterns and functional signatures [22]. Sources for GeneAnalytics™ include Reactome, WikiPathways, R&D Systems, GeneGo, and PubChem.

### Quantitative reverse transcription polymerase chain reaction (RT-qPCR)

Calu-3/2B4 cells either infected with SARS-CoV-2 WA-1 or Omicron were harvested 24 hours post infection to measure the expression of *BST2*, *ZBP1*, *CXCL11*, and *IFITM1* through RT-qPCR. cDNA samples were prepared as previous described and amplified using specific primer sets (S1 Table) and Bio-Rad iTaq™ Universal SYBR Green SuperMix kit. BioRad CFX96 Real-Time PCR Thermal Cycler was used to perform RT-qPCR with the thermal cycling program: 95°C for 3 minutes, 95°C for 10 seconds, and 58°C for 30 seconds for 39 cycles each step. 18S rRNA level was used as internal control.

### Creation of graphs and statistical analysis

Graphs were created and statistical testing was performed in GraphPad Prism 10.0.0. Volcano plots and heatmaps were created using the public server at usegalaxy.org [23].

## Results

### SARS-CoV-1 and SARS-CoV-2 productively infect Calu-3/2B4 cells

Calu-3/2B4 cells are a non-ciliated human bronchial epithelial cell with more intense and stable membrane expression of human ACE2, the entry receptor for both SARS-CoV-1 and SARS-CoV-2 [24]. Therefore, Calu-3/2B4 cells provide a more consistent cell culture system for studying SARS-CoV-1 and -2 infection. In our previous study, Calu-3/2B4 cells showed high susceptibility to SARS-CoV-1 infection and supported rapid viral replication compared with original Calu-3 cells [9]. Since SARS-CoV-2 also exhibits similar tissue tropism in the lower respiratory tract, infection with Calu-3/2B4 cells may provide insights into clinical outcomes relevant to human pathology.

**Fig 1:**
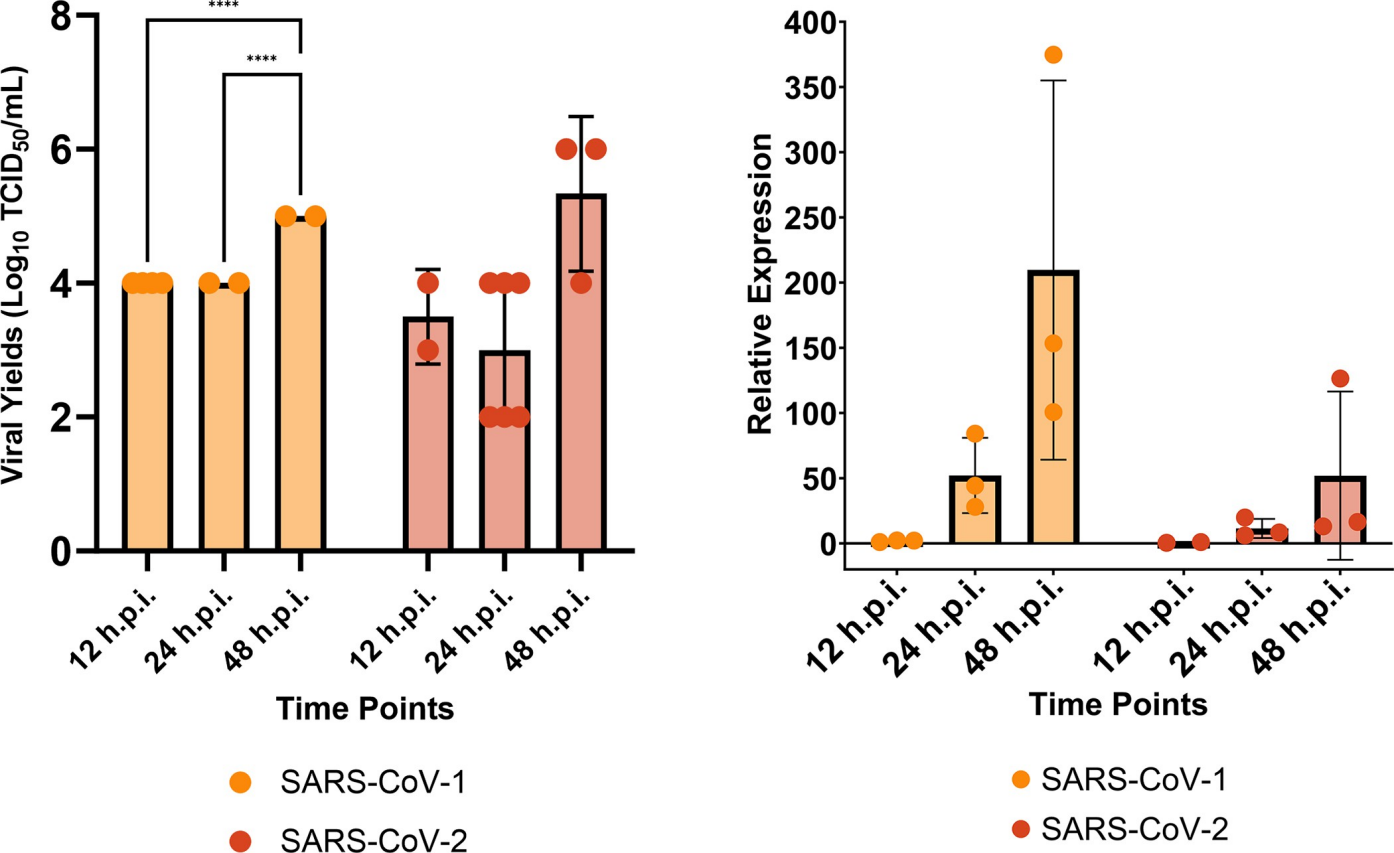
SARS-CoV-1 and -2 productively replicate in Calu-3/2B4s over time. Calu-3/2B4 cells were infected with either SARS-CoV-1 or SARS-CoV-2 (WA-1) at a MOI of 1.0 in 6-well plates. Cell-free supernatant and infected cells were collected at 12-, 24- and 48-hpi, and the **(A)** viral yield and **(B)** viral genome fold (relative to 18S) of SARS-CoV-1 and SARS-CoV-2 were measured through TCID_50_ assay and RT-qPCR, respectively. Mixed effects analysis with Tukey’s multiple comparisons was used for statistical analysis. No comparisons indicate insignificant differences. **** p < 0.0001.

Therefore, we first validated the kinetics of these viruses’ replication infection in these cells. Following internal optimization, we selected a MOI of 1.0 to ensure productive replication of SARS-CoV-1 and -2 in Calu-3/2B4 cells. Calu-3/2B4 cells were infected with SARS-CoV-1 and -2 at a multiplicity of infection (MOI) of 1.0, and then harvested the cell supernatant at designated time points to determine the yields of infectious progeny virus At 12 hours post-infection (hpi), there was infectious progeny virus titer at 4 (SARS-CoV-1) and 3.5 (SARS-CoV-2) log_10_ TCID_50_/mL (Fig 1A). At 24 hpi, it remained at the same levels for SARS-CoV-1, but for SARS-CoV-2, viral levels slightly decreased to 3 log_10_ TCID_50_/mL. At 48 hpi, SARS-CoV-1 viral yields were significantly higher at 5 log_10_ TCID_50_/mL, while SARS-CoV-2 had 5.3 log_10_ TCID_50_/mL. Similar trends were observed in both SARS-CoV-1 and SARS-CoV-2 viral genome replication, with rapid replication kinetics within 12 to 48 hpi (Fig 1B). However, at 48 hpi, SARS-CoV-1 showed a higher genome fold increase than SARS-CoV-2.

Our results show that Calu-3/2B4 cells are productively infected by both SARS-CoVs, with highest titers seen at 48 hpi. Furthermore, SARS-CoV-2 has comparable replication kinetics with SARS-CoV-1. With the proven susceptibility of Calu-3/2B4 cells to both SARS-CoV-1 and -2, we then performed RNA-Seq to characterize the innate immune response over time to both β-CoVs infection.

### SARS-CoV-1 infection induces expression of pattern recognition receptors, chemokines, and interferon-stimulated genes

**Fig 2:**
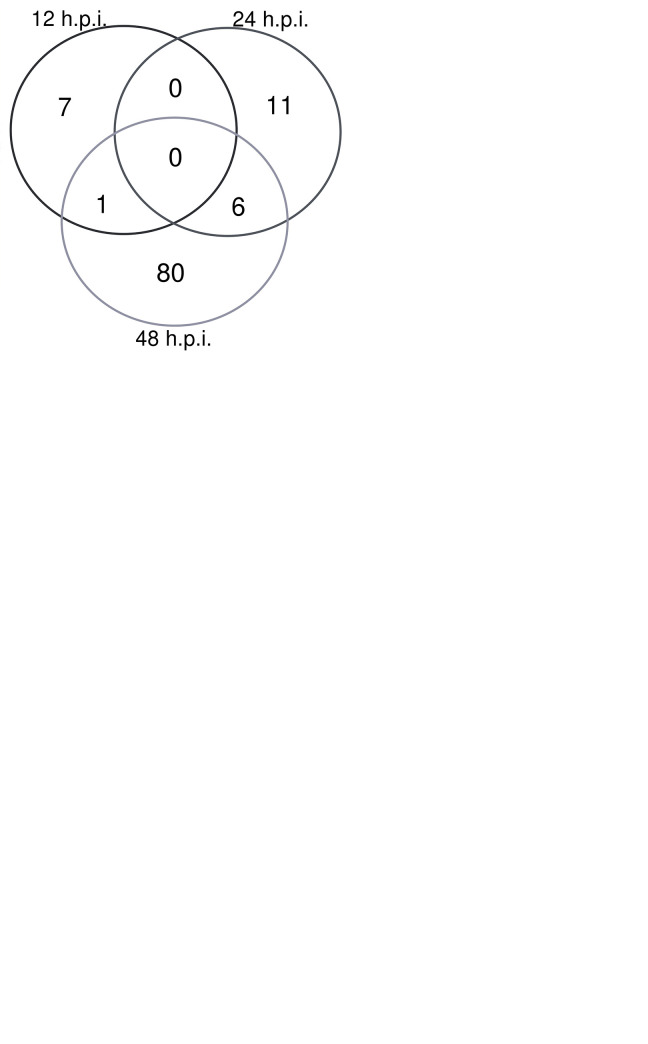
SARS-CoV-1 infection leads to differential expression of genes at indicated time points. Volcano plots were utilized to visualize DEGs in SARS-CoV-1 and mock-infected Calu-3/2B4 cells after **(A)** 12 hpi, **(B)** 24 hpi, and **(C)** 48 hpi. Genes were included in the volcano plot with a log2foldchange value <-0.585 or >0.585 and p-value < 0.05. **(D)** Venn diagram of the number of DEGs at multiple time points during SARS-CoV-1 infection. DEGs were included if their log_2_fold change value was less or more than ± 0.585 and had a p_adj_-value of < 0.05. **(E)** After selecting canonical pathways in IPA, the number of genes at each time point was again visualized, and those that were found at multiple time points were further explored through ClueGO.

We first compared the RNA-Seq results of SARS-CoV-1 versus mock-infected Calu-3/2B4 cells to systematically explore differentially expressed genes (DEGs) and pathways. As shown in Fig 2A–2C, we note that a total of 6, 37, and 357 genes were differentially regulated at 12-, 24-, and 48-hours respectively post-SARS-CoV-1 infection. As seen in the volcano plots, all DEGs were significantly downregulated at 12 hpi (Fig 2A). However, at 24 hpi, DEGs were only upregulated (Fig 2B). At 48 hpi, 299 genes were upregulated, though 58 were downregulated as well (Fig 2C).

We then focused on DEGs that showed sustained changes at multiple time points as potential targets of interest against SARS-CoV-1 infection (Fig 2D). Among these DEGs, two (*BIRC3* and *CXCL8*) were downregulated at 12 hpi, then upregulated at 48 hpi. Thirty-five DEGs including *RIG-I*, members of the 2’-5’ oligoadenylate synthetases (*OAS*) family, and members of the IFN-induced protein with tetratricopeptide repeats family (*IFIT*) were upregulated at both 24 and 48 hpi. This indicates the activation of the innate immune regulators and genes later in the time course following SARS-CoV-1 infection in pathologically relevant human lung epithelial cells.

Afterwards, genes with a log_2_fold scale of less or more than ± 1.5 and a p_adj_-value of ≤ 0.05 were inputted into IPA to annotate their corresponding pathways [6]. Canonical pathways were then selected; examples include IL-10 signaling, the role of pattern recognition receptors in recognition of bacteria and viruses, coronavirus replication pathway, and activation of IRF by cytosolic pattern recognition receptors (S2 Table). After extracting genes that were part of these pathways, we found seven genes differentially expressed at multiple time points between SARS-CoV-1- and mock-infected cells: *CCR1*, at 12- and 48-hpi, and *CCL22*, *CXCL10*, *CXCL11*, *OAS2*, *IFNB1*, and *DRD1* at 24- and 48-hpi (Fig 2E).

Following IPA analysis and to further validate our results, we performed functional enrichment analysis using the Cytoscape App ClueGO on these seven genes differentially expressed at multiple time points. We found that 62.5% of the terms found were related to chemokine receptors binding to chemokines, 33.3% of the terms were related to positive regulation of calcium ion transport, and 4.2% of the terms were found in the Toll-like receptor signaling pathway, another type of PRRs. The highest percentage of genes were found in the term “Chemokine receptors bind chemokines”. Therefore, it appears that chemokines are an important part of the host cell’s defense against SARS-CoV-1 infection, which supports previous findings [9,25].

### SARS-CoV-2 infection activates immune response pathways and genes in order to restrict viral replication

**Fig 3:**
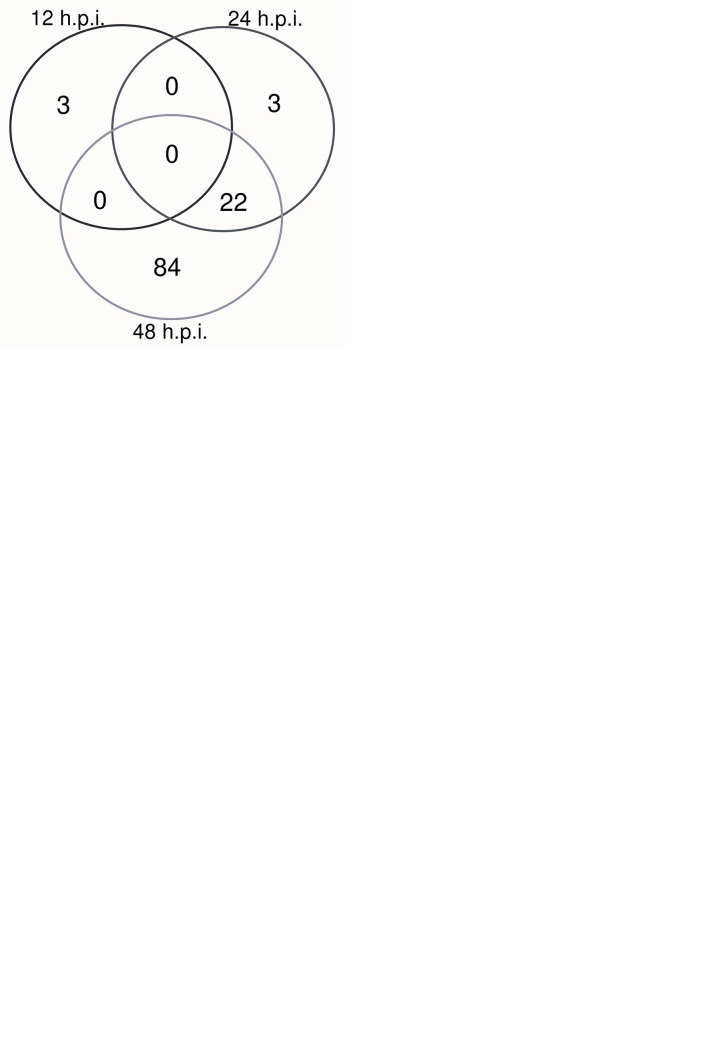
SARS-CoV-2 infection triggers increasing gene expression over time. Volcano plots were utilized to visualize DEGs in SARS-CoV-2 and mock-infected Calu-3/2B4 cells after **(A)** 12 hpi, **(B)** 24 hpi, and **(C)** 48 hpi. Genes were included in the volcano plot with a log2foldchange value < -0.585 or >0.585 and p-value < 0.05. **(D)** After selecting for DEGs with log_2_fold change value<-0.585 or >0.585 and a p_adj_-value of < 0.05, a Venn diagram of the number of DEGs during SARS-CoV-2 infection was created to visualize overlapping gene expression. **(E)** After canonical pathways were selected in IPA, the number of genes at each time point was seen in the Venn Diagram, and genes found at multiple time points were further characterized through ClueGO.

To gain a general understanding of the effects of SARS-CoV-2 infection, we selected for DEGs (log_2_fold change value < -0.585 or > 0.585 and p_adj_-value of < 0.05) in SARS-CoV-2- compared to mock-infected cells. There was a total of 878 DEGs, and at 12 hpi, 17 genes were significantly downregulated, while three were upregulated (Fig 3A). Similar to what was seen in SARS-CoV-1 compared to mock-infected Calu-3/2B4s, 34 DEGs were significantly upregulated at 24 hpi (Fig 3B). At 48 hpi, 308 genes were differentially downregulated, while 556 were upregulated for a total of 864 genes (Fig 3C). At 12- and 48-hpi, six genes were differentially expressed during SARS-CoV-2 infection (*ICAM1*, *TNFAIP3*, *CXCL8*, *BIRC3*, *RELB*, and *TRIM31)*, while 34 were differentially regulated at both 24- and 48-hpi (Fig 3D). This highlights the time-dependent differences in gene activation following SARS-CoV-2 infection.

After inputting genes with a log_2_fold scale of less than -1.5 or more than 1.5 and a p_adj_-value of ≤ 0.05 into IPA, we then examined canonical pathways related to infectious diseases and immunity [6]. Examples of pathways that were selected included: coronavirus pathogenesis, IFN signaling, the role of RIG-I-like receptors (RLRs) in antiviral innate immunity, and communication between innate and adaptive immune cells (S3 Table). Similar canonical pathways found in SARS-CoV-1-infected cells were also selected for SARS-CoV-2-infected cells, such as NF-κB activation by viruses, JAK/STAT signaling, and coronavirus pathogenesis pathway.

When we extracted the genes that were part of these canonical pathways, we found that 22 DEGs are expressed at 24- and 48-hpi (Fig 3E). We then inputted these 22 DEGs into ClueGO. After functional enrichment analysis, approximately 44% of the terms were related to the negative regulation of viral genome replication, demonstrating how ISGs may act to restrict SARS-CoV-2 infection. IFN alpha/beta signaling and “response to Type I IFN” pathways composed 14.0% and 9.3% of the terms, respectively, signifying activation of the type I IFN response. Response to viral infection also occurs, as seen in the pathways “Response to virus” (7.0%) and “Cellular response to virus” (11.6%). These results demonstrate that the innate immune response is functioning through multiple pathways to restrict SARS-CoV-2 infection in Calu-3/2B4s over time.

### SARS-CoV-2 infection induces more robust IFNs signaling than SARS-CoV-1 in Calu-3/2B4 cells

**Fig 4:**
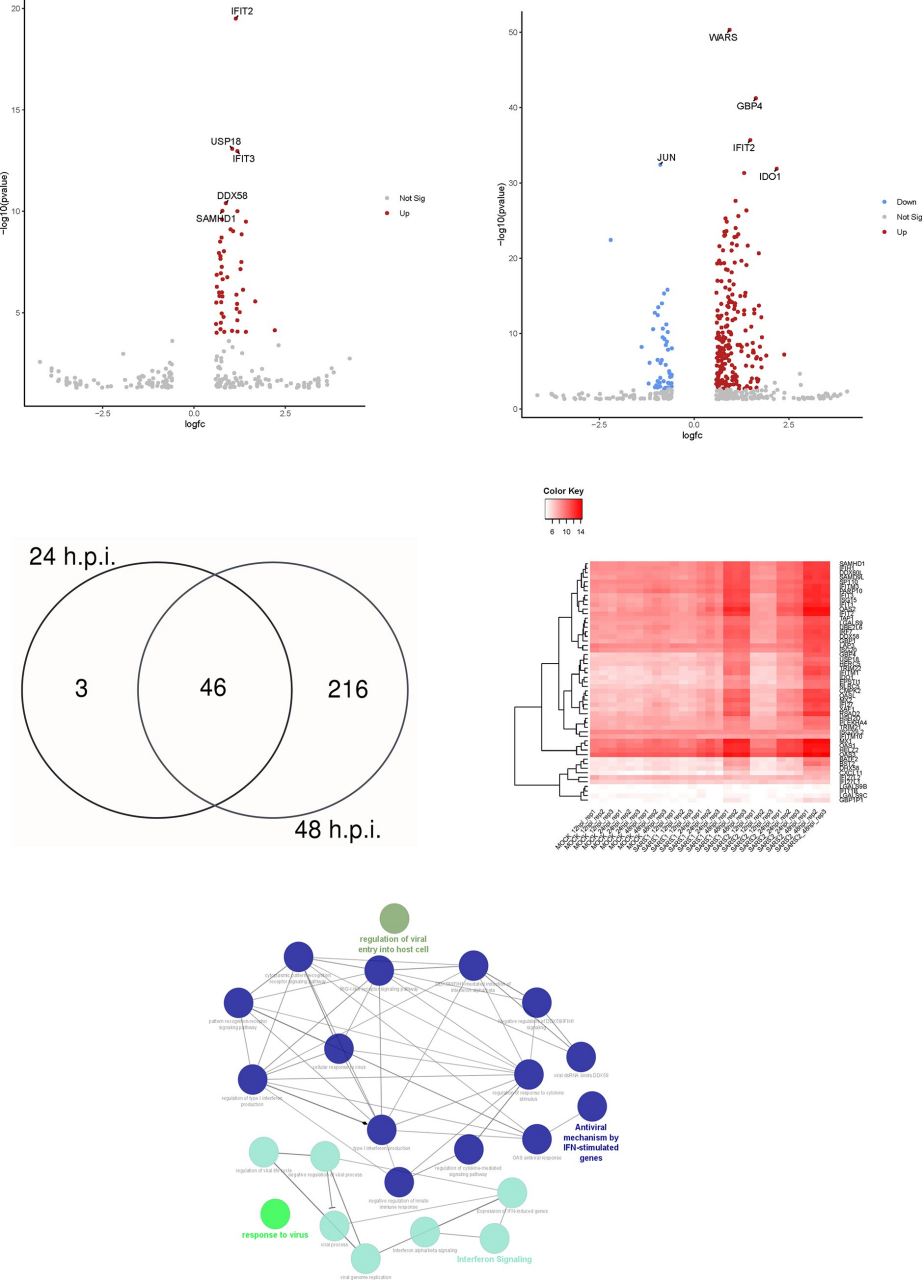
DEGs induced by SARS-CoV-2 and -1 were compared to find similarities and broadly characterize functionally enriched pathways during infection. Volcano plots were utilized to visualize DEGs in SARS-CoV-2 and SARS-CoV-1-infected Calu-3/2B4 cells after **(A)** 24 hpi and **(B)** 48 hpi. Genes were included in the volcano plot with a log2foldchange value of <-0.585 or >0.585 and p-value < 0.05. **(C)** After selecting for DEGs with log_2_fold change values <-0.585 or >0.585 and a p_adj_-value of < 0.05, a Venn diagram was created to determine the number of genes that significantly differed between SARS-CoV-1 and -2 infection at multiple time points. **(D)** A heat map of the 46 genes differentially expressed at 24 and 48 hpi was created. Genes were hierarchically clustered by infection status and time point to visualize expression over time. **(E)** Functionally enriched pathways elicited by the 46 genes were regulation of viral entry into host cell, response to virus, interferon signaling, and antiviral mechanism by IFN-stimulated genes.

To directly compare the similarities in the innate immune response to viral infection and select potential host targets against β-CoVs, we examined genes that were differentially expressed (log_2_fold change value < -0.585 or >0.585 and p_adj_-value of < 0.05) in response to SARS-CoV-2 compared to SARS-CoV-1 infection. There were no significantly expressed genes at 12 hpi. At 24 hpi, there were 49 genes which were enhanced in SARS-CoV-2-infected compared to SARS-CoV-1-infected Calu-3/2B4 cells (Fig 4A). For example, expression of genes encoding for *DDX58/RIG-I*, *USP18*, *SAMHD1*, and several members within the *IFIT* family was greater in response to SARS-CoV-2 than SARS-CoV-1 infection. At 48 hpi, there were 48 genes more highly expressed in SARS-CoV-1-infected cells, like *JUN*, a gene expression regulator, *HSPA6*, which is involved in enzyme binding, and *FOSB*, which regulates cell proliferation, differentiation, and transformation. However, 214 DEGs have greater expressions in SARS-CoV-2-infected than those of SARS-CoV-1-infected cells (Fig 4B).

Using the criteria of log_2_foldchange < -0.585 or >0.585 (or fold change of <-1.5 or >1.5) and a p_adj_-value of 0.05 (for statistical significance), we then identified 46 genes which have significantly upregulated gene expression at 24 and 48 hpi in SARS-CoV-2 infected cells than SARS-CoV-1 infected cells (Fig 4C). Among these DEGs, many are involved either in regulating the innate inflammatory and/or antiviral processes, such as *CXCL11*, or ISGs like IFN-induced protein with tetratricopeptide repeats (*IFIT*), myxovirus resistance (*Mx*), and *OAS*s. Their differential expressions at each time point evaluated among mock-, SARS-CoV-1-, and SARS-CoV-2-infected cells can be visualized (Fig 4D). The heat map shows that many genes are differentially expressed over time, with greatest upregulation at 48 hpi in both SARS-CoV-1 and -2 infection. Using ClueGO, we found that these differentially regulated genes are functionally enriched in the following pathways: regulation of viral entry into the host cell (5.6%), antiviral mechanism by ISGs (28.9%), IFN signaling (33.3%), and response to virus (32.2%) (Fig 4E). Specifically, identified sub-pathways generally involved the activation of PRRs, including the RLRs signaling pathway. Genes encoding Type I IFNs were also induced, which may lead to the activation of ISGs to regulate viral processes and replication negatively. Additionally, more than half of the genes regulate IFN-related signaling pathways, highlighting the activation of the innate immune response. Because these genes were all significantly upregulated in both SARS-CoV-1 and -2 infection, especially at the final time point measured, they were of interest as targets for host-directed antivirals.

#### Four genes are identified as targets for potential host-directed antivirals

To further select potential genes as targets against β−CoVs, we chose DEGs whose expression levels were less or greater than a log_2_fold scale of ± 1.5 and had a p-value of < 0.05 for statistical significance and further analysis [6]. IPA was then utilized to annotate those selected DEGs to pathways related to infectious diseases and immunity, and subsequently inputted those genes into ClueGO for functional enrichment analysis. To this end, IPA analysis successfully selected key canonical pathways related to infectious diseases and immunity at 12-, 24-, and 48-hpi (S4 Table). Specifically, canonical pathways identified following SARS-CoV-1 and -2 infection after 48 hours include IFN signaling, coronavirus replication pathway, role of PRRs in recognition of bacteria and viruses, coronavirus pathogenesis pathway, activation of interferon regulatory factor (IRF) by cytosolic PRRs, and JAK/STAT signaling.

**Fig 5:**
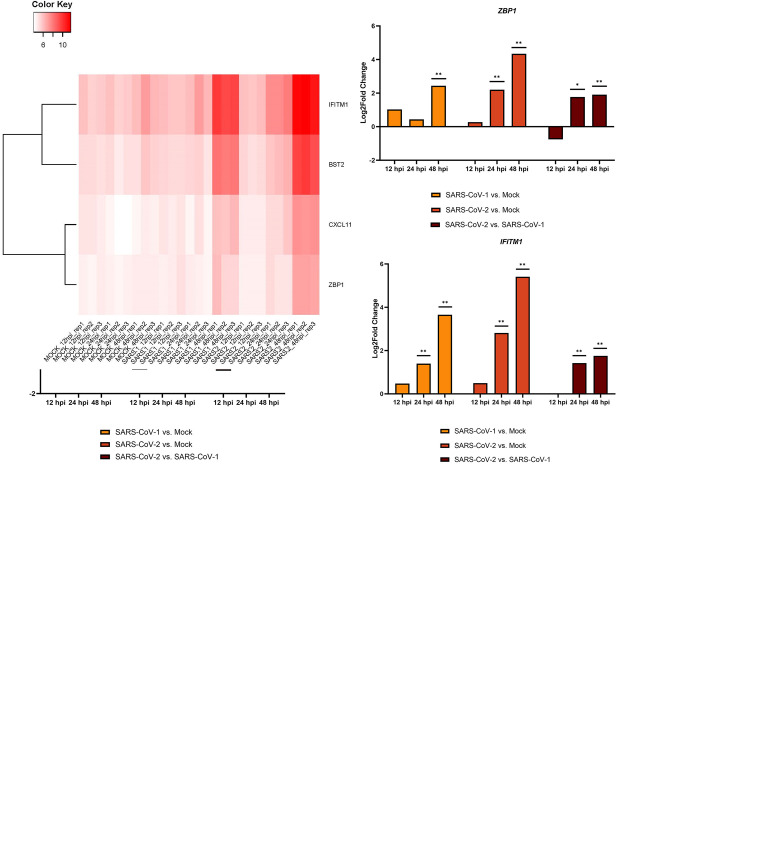
Genes of interest (*BST2*, *ZBP1*, *CXCL11*, and *IFITM1)* demonstrate increased expression over time. **(A)** Selection of two genes of interest based on expression at both 24- and 48-hpi in SARS-CoV-1- and -2-infected cells after IPA. **(B)** Heatmap of genes of interest shows highest expression at 48 hours following infection with either β-CoV. **(C)** Log_2_fold change values of the genes of interest at the indicated time points and using the comparisons from RNA-Seq demonstrate increased expression over time and generally higher expression in SARS-CoV-2- compared to SARS-CoV-1-infected cells. * p < 0.05; ** p < 0.01.

From the selected canonical pathways, we chose DEGs showing overlapping expression at multiple time points in SARS-CoV-2- and -1-infected cells as “genes of interest” for further exploration. When focusing on the genes detected during SARS-CoV-1 and -2 infection, we found that at 24 and 48 hpi, two ISGs, i.e., bone marrow stromal cell antigen 2 (*BST2*, also known as *CD317*, *HM1*.*24*, and *TETHERIN*) and Z-DNA binding protein 1 (*ZBP1*, also known as *DAI*, *DLM1*, *DLM-1*, and C20orf183), could be identified in canonical pathways related to immunity and infectious diseases (Fig 5A). Finalizing the selection of genes of interest, we initially compared those genes selected based on the less-stringent criteria (log_2_foldchange < -0.585 or > 0.585 and p_adj_-value < 0.05) with those related to immunity and infectious diseases. The following gene candidates thus emerged, including CXC motif chemokine ligand 11 (*CXCL11*, also known as *IP9*, *H174*, *IP-9*, *b-R1*, *I-TAC*, *SCYB11*, or *SCYB9B*), interferon-induced transmembrane protein 1 (*IFITM1*, also known as *9–27*, *CD225*, *IFI17*, *LEU13*, or *DSPA2a*), along with *BST2*.

To ensure that *BST2*, *ZBP1*, *CXCL11*, and *IFITM1* were appropriate for both β-CoVs infection, we further analyzed the expression of selected genes of interest. The heatmap shows that highest expressions were at 48 hpi in both SARS-CoV-1- and SARS-CoV-2-infected Calu-3/2B4 cells (Fig 5B). Generally, the RNA-Seq data demonstrate that the expressions of these four genes were upregulated over time as compared those of mock-infected cells (Fig 5C). Significant differences in gene expression compared to mock usually began at 24 hpi and continued at 48 hpi. At 24- and 48-hpi, the genes of interest were significantly greater in SARS-CoV-2- compared to SARS-CoV-1-infected Calu-3/2B4s. Therefore, these four genes appear to respond to both β-CoVs infection, and they may play a greater role in protecting the host against SARS-CoV-2 infection.

As further verification, when we reviewed the list of genes identified from IPA, we found that *CXCL11* was found at both 24- and 48-hours post-SARS-CoV-1-infection. *BST2*, *ZBP1*, and *IFITM1* emerged at 48 hpi. In SARS-CoV-2-infected cells, even when using the criteria of log2foldchange ± 0.585 and p_adj_ < 0.05, *BST2*, *IFITM1*, and *CXCL11* were found at 24 hpi, and all four genes were identified at 48 hpi. All four genes are identified at 48 hpi following IPA when comparing host response induced by SARS-CoV-1 and SARS-CoV-2 infection. Therefore, though these four genes are more highly and significantly induced by SARS-CoV-2 infection, SARS-CoV-1 infection is still able to activate their expression, making them genes of interest in viral infection.

When performing further analysis, functional enrichment and ClueGO analyses revealed that these four genes belong to two key pathways: negative regulation of the viral life cycle and response to type I IFN. Other pathways related to these two pathways included regulation of the viral process, regulation of viral genome replication, regulation of the viral life cycle, and expression of ISGs. To further validate our findings, we utilized GeneAnalytics™. Through the LifeMap Science’s Gene Cards suite, we connected genes of interest to pathways and diseases. Based on this tool of gene analysis, we found a total of 68 GO terms could be associated with these four selected genes of interest. *BST2*, *ZBP1*, *CXCL11*, and *IFITM1* were involved in protein binding, and they are also broadly engaged in biological processes related to IFNs and responding to infections. *BST2*, *ZBP1*, and *IFITM1* were involved in the defense response, the innate immune response, and the immune system process; these findings corroborated our results seen in ClueGO.

We then explored GeneAnalytics™ Pathways, specifically SuperPaths, which aggregates twelve pathway sources to create lists of pathways and genes part of these pathways [22]. A total of twenty pathways were identified, and we selected seven pathways for further investigation. In the “SARS-CoV-2 Infection” SuperPath, Reactome identified *BST2* and *ZBP1*, which supports the role of these genes in SARS-CoV-2 infection. In the “SARS-CoV-2 Innate Immunity Evasion and Cell-specific Immune Response” SuperPath, *IFITM1* was found to be a part of it through WikPathways. Interestingly, Reactome identified *ZBP1* as a “Potential Therapeutic For SARS,” and *BST2* was part of the “SARS-CoV-1-Host Interactions” SuperPath. The results demonstrate that these genes have been identified in both β-CoVs infection, further supporting our interest in exploring them as targets.

#### Omicron BA.1 activates the genes of interest at 24 hpi, supporting their potential as antiviral targets

**Fig 6:**
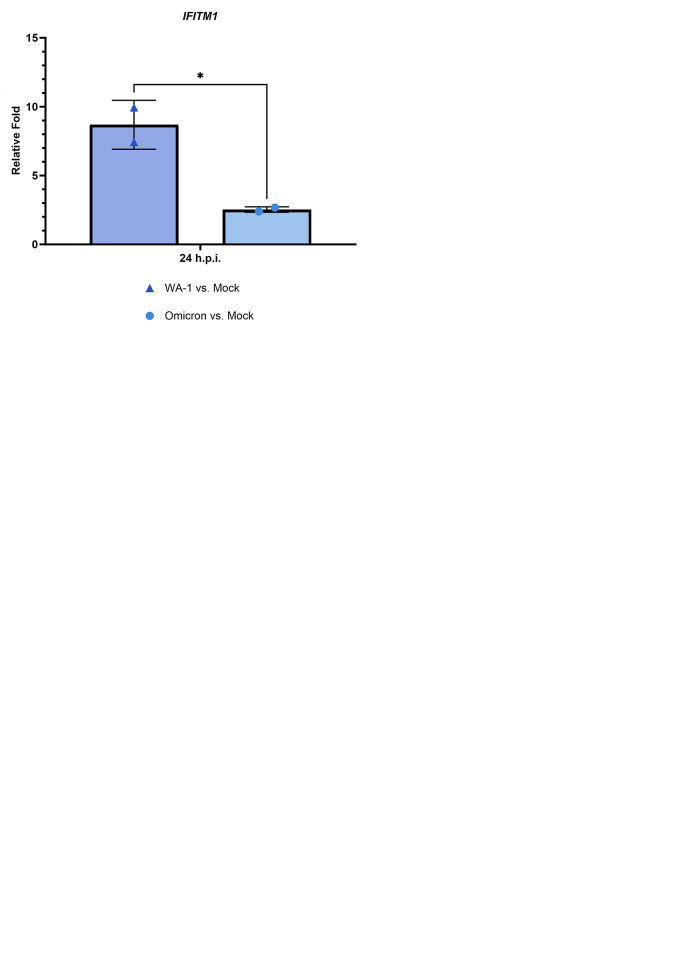
Though SARS-CoV-2 WA-1 and Omicron BA.1 have similar viral yields at 24 hpi, genes of interest are more highly activated by the USA-WA1/2020 strain. **(A)** Calu-3/2B4 cells were infected with SARS-CoV-2 USA1/2020 strain or Omicron BA.1 at a MOI of 1.0 in 6-well plates. Cell-free supernatant was collected at 24 hpi for viral titration using the TCID_50_ assay. **(B)** *BST2*, **(C)** *ZBP1*, **(D)** *CXCL11*, and **(E)** *IFITM1* were more highly induced by SARS-CoV-2 WA-1 compared to Omicron BA.1. at 24 hpi. Statistical significance was determined through t-tests. No significance unless otherwise shown as * p < 0.05.

As variants of concern have circulated throughout the pandemic, we were interested in determining whether they would trigger expression of the genes of interest as well. Therefore, Calu-3/2B4 cells were infected with SARS-CoV-2 WA-1 or Omicron BA.1 at a MOI of 1.0, and cell-free supernatant and cellular RNA were extracted at 24 hpi. The average titer for the SARS-CoV-2 USA-WA1/2020 strain was 6.7 log_10_ TCID_50_/mL, while Omicron BA.1’s was lower at 5.4 log_10_ TCID_50_/mL (Fig 6A). Therefore, these results indicate that Omicron BA.1 has a relatively slower replication kinetic compared to WA.1.

When we measured expression of our genes of interest, *BST2* had a 4.9-fold increase in WA-1- compared to mock-infected cells and a 1.8-fold increase in Omicron BA.1- compared to mock-infected cells (Fig 6B). For *ZBP1*, at 24 hpi, expression in WA-1- compared to mock-infected cells was 7.7-fold higher, and it was 3.2-fold higher in Omicron BA.1- compared to mock-infected Calu-3/2B4s (Fig 6C). *CXCL11*’s expression is significantly higher in WA-1- versus mock-infected cells (13.2-fold) than Omicron BA.1- versus mock-infected cells (2.3-fold) (Fig 6D). *IFITM1* expression is also significantly greater in WA-1- versus mock-infected cells (8.7-fold) compared to Omicron BA.1- versus mock-infected cells (2.5-fold) (Fig 6E). Therefore, our results showed that infection with either WA-1 or Omicron BA.1 activates the genes of interest, supporting our interest in utilizing these genes in antiviral β-CoVs treatment.

## Discussion

In the past twenty years, three highly pathogenic β-CoVs, i.e., SARS-CoV-1, MERS-CoV, and SARS-CoV-2, have emerged, causing significant morbidity and mortality worldwide. Though these highly pathogenic β-CoVs have demonstrated their ability to disrupt our social, economic, and public health worldwide, there are currently no licensed medical countermeasures (MCMs) that can treat all β-CoVs. Despite the existence of a few effective MCMs for COVID-19, efforts to identify new druggable targets, especially those derived from hosts, against pan β−CoVs to address unmet medical challenges remain urgent. Under this general premise, we characterized and compared the host innate immune responses against SARS-CoV-1 and SARS-CoV-2 by using pathologically relevant human bronchial epithelial cells, aiming to identify host antiviral molecules as potential targets for future drug development against at least both SARS-CoV-1 and -2.

To understand how the innate immune response plays a role in the response to SARS-CoV-1- and -2, we initially compared the permissiveness and kinetics of Calu-3/2B4 cells between SARS-CoV-1 and SARS-CoV-2, followed by assessing their corresponding temporal and global host responses based on RNA-Seq analysis. We noted that Calu-3/2B4 cells showed a near-equal permissiveness to SARS-CoV-1 and SARS-CoV-2 infection, leading to similar viral yields, and resulting in the induction of signaling cascades directly related to innate antiviral immune responses. Specifically, innate antiviral responses, especially those caused by the “RLRs-IFNs” axis-mediated signaling cascades, were highly upregulated to counteract viral infection. Those significantly upregulated genes identified could be divided into four non-mutually exclusive categories: IFN signaling, response to virus, antiviral mechanism by ISGs, and regulation of viral entry into host cell.

To identify potential targets for MCM development, we narrowed down four genes for further examination: *BST2*, *ZBP1*, *CXCL11*, and *IFITM1*. The genes work in various ways to inhibit viral replication, both directly (*BST2* and *IFITM1*) and indirectly (*ZBP1* and *CXCL11*). As a transmembrane protein, *BST2* blocks the release of enveloped viruses by tethering virions, limiting replication, and it has previously been shown to restrict the release of SARS-CoV-1 and SARS-CoV-2 [26–28]. *BST2* is also targeted by viral proteins to block its action, suggesting its significance in the innate immune response. *ZBP1* is part of the PANoptosis death signaling pathway and binds to foreign DNA [29]. It is upregulated by the type I IFN receptor and can activate immune cells during SARS-CoV-2 infection [30]. Importantly, elevated expression of *ZBP1* has been reported in patients suffering from severe COVID-19, thereby suggesting that it might be involved in the pathogenesis of SARS-CoV-2 [31]. *CXCL11* is induced by Type II IFNs, and this pro-inflammatory gene acts in an autocrine and paracrine manner on various epithelial, endothelial, and immune cells [32]. Higher expression of *CXCL11* is seen in permissive hosts infected by β-CoVs, including Calu-3 cells and COVID-19 patients, and it promotes the migration of immune cells to the lungs following infection [25,32]. Lastly, *IFITM1* restricts viral cellular entry by targeting the spike protein [30,33]. As this gene is localized at the plasma membrane, it may possibly block the merger of the viral and cellular membrane [33]. While the role of *IFITM1* in inhibiting viral replication has been well documented *in vitro*, many β-CoVs have evolved sophisticated strategies to hijack its induction, suggesting its importance in the host defense against CoVs [33].

We also examined the broader relevance of these genes in other diseases, and through GeneAnalytics™, we found a total of 57 diseases in which the genes of interest were associated with. There were three infectious diseases that included three of the genes of interest: hepatitis C, human immunodeficiency virus (HIV) type 1, and influenza. Interestingly, hepatitis C virus had also been identified in ClueGO when comparing SARS-CoV-2 and mock-infected cells. *CXCL11* and *BST2* were differentially expressed in the liver, while *IFITM1* was also predicted to be expressed by GeneCards. Influenza, a respiratory illness like SARS-CoV-1 and -2, was expected to lead to differential expression of *BST2*, *IFITM1*, and *ZBP1*. Through Gene Analytics™, *ZBP1* was found to be differentially expressed in blood, further supporting its association with influenza. Overall, we found that *BST2*, *ZBP1*, *CXCL11*, and *IFITM1* are associated with infection with SARS-CoV-1, SARS-CoV-2 WA-1 and Omicron BA.1, hepatitis C virus, HIV, and influenza virus. These results hint at the broader applications of these genes, reinforcing their potential as targets for host-directed antivirals.

Our findings were similar to a previous publication comparing SARS-CoV-2, SARS-CoV, MERS-CoV, and HCoV-229E, as three of the four genes (*BST2*, *ZBP1*, and *CXCL11*) were identified as having higher expression in SARS-CoV-2-infected cells compared to the other coronaviruses [13]. A separate group found that *BST2* is involved in suppressing SARS-CoV-2 replication *in vitro*, and *ZBP1* can decrease the replication of SARS-CoV-1, SARS-CoV-2, influenza, and other viruses [28]. Supporting the importance of these genes, a recent study demonstrated that in Caco-2 cells, human colon cancer cells, knockout of *BST2* led to increased SARS-CoV-2 dissemination and replication, reinforcing the importance of this gene in restricting viral infection [34]. *IFITM1* overexpression suppressed other human coronaviruses such as hCoV 229E and variants of concern, but in human lung cells, knockdown of this gene led to similar levels of SARS-CoV-2 variants of concern as control [35–37].

IPA also highlighted the following upstream regulators of these four genes: STAT1, IFN-alpha, IFN-beta, and poly rI:rC-RNA. This indicates that these genes of interest are activated through IFN signaling or foreign RNA. Thus, another potential mechanism to investigate may be IFNs, especially because β-CoVs are well-known to delay activation of IFNs [12]. Thus, IFNs have been investigated as a potential therapeutic against COVID-19 with mixed results [38]. Taken together, the results emphasize the critical role that these genes and the innate immune response plays during the course of viral infection.

There are multiple future directions for our study. Although β-CoVs are generally characterized as a respiratory disease, COVID-19 patients have reported neurological signs and symptoms such as headache and fatigue [39]. Supporting the effects of β-CoVs on the nervous system, several neurological pathways were identified through IPA, such as the neuroinflammation signaling pathway, when comparing SARS-CoV-1 and -2-infection.

In regard to the genes, our next step would be to knockout and overexpress *BST2*, *ZBP1*, *CXCL11*, and *IFITM1* in our pathologically relevant human lung epithelial cells to aid in the development of potential therapeutic targets against β-CoVs. In addition, we will look in further probing the mechanistic and functional importance of these genes and their pathways in SARS-CoV-1 and -2 infection in both the short- and long-term immune response. Examining how other variants of concerns such as Omicron BA.2 interact with these genes would be of interest.

Clinically, our goal would be to develop host-directed antivirals that target these genes and promote a successful innate immune response in β-CoVs infection. For example, losartan, which is an angiotensin II receptor blocker, upregulates both *BST2* and *IFITM1* in human kidney cells [40]. However, following clinical trials, losartan was not recommended for COVID-19 patients [41]. This highlights the difficulty and importance of identifying small molecules, biologics, and gene therapies against β-CoVs infection; thus, more research is needed to achieve this goal.

This is one of the first studies to directly compare the temporal and global gene expression profiles of SARS-CoV-1 and SARS-CoV-2 in pathologically relevant human lung epithelial cells. These β-CoVs can delay activation of the innate immune response and ISGs, enabling them to establish early infection in the host. Therefore, subverting β-CoVs’ ability to evade innate antiviral responses to prevent exacerbated inflammatory responses and even long COVID is intriguing. Though the innate immune response has been heavily examined in β-CoVs, we emphasize the importance of exploring host innate responses against SARS-CoV-1 versus SARS-CoV-2 to better develop effective MCMs against past, current, and future β-CoVs. In light of our analysis and existing literature, we believe the innate immune response plays an important, but not yet fully defined, role in keeping SARS-CoV-1 or -2 infection and subsequent pathogenesis in check.

## Supporting information

S1 Table Primer sequences for RT-qPCR. All primers were purchased from Integrated DNA Technologies.
(XLSX)

S2 Table SARS-CoV-1 vs Mock–IPA Tables. Canonical pathways and genes selected following IPA analysis at 12, 24, and 48 hpi. Genes found at multiple time points (“Overlapping Genes”) were then extracted.
(XLSX)

S3 Table SARS-CoV-2 vs Mock–IPA Tables. Canonical pathways and genes selected following IPA analysis at 12, 24, and 48 hpi. Genes found at multiple time points (“Overlapping Genes”) were then extracted.
(XLSX)

S4 Table SARS-CoV-2 vs SARS-CoV-1 –IPA Tables. Canonical pathways and genes selected following IPA analysis at 12, 24, and 48 hpi. Genes found at multiple time points (“Overlapping Genes”) were then extracted.
(XLSX)
